# Reinstatement of Drs. Heath and May

**Published:** 1902-08

**Authors:** 


### Reinstatement of Drs. Heath and May.

AFTER two months' suspension on an unproved accusation these two capable officers of the health department will return to duty August 2, 1902. The history of this affair shows how much easier it is to assert than to prove. That a school principal should be careless of his tongue is without excuse; such an official should be a model in word and deed. Would it not be in order now for Superintendent Emerson to administer a reprimand to Principal Knell? And, yet, perhaps the opposition to vaccination is responsible for much immoderate language; if so it is better to pity than to scold such ignorance.

The action of the health commissioner is deserving of all praise. He was somewhat overcareful, perhaps, in delaying so long, but his final statement in rendering his decision, makes the vindication of the accused physicians so complete that their prolonged suspension can be regarded with complacency. We congratulate Drs. May, Heath, and Greene on the satisfactory termination of a disagreeable trial.

---

IN Alabama it has been decided that osteopaths must take the regular examination of the state board. The *Alabama Medical*

*Journal*, July, 1902, publishes the following reference to the action of the courts:

We publish in this issue of the Journal the decision handed down by the Supreme Court of Alabama, which is a very important case with reference to osteopathy. Commenting upon this decision, the *Journal of the American Medical Association* says:

By a decision of the Supreme Court, rendered June 28, in the case of Eugene Bragg vs. the State of Alabama, the decision of the lower court was affirmed. The decision affects several hundred men throughout the state, and will have the virtual effect of driving them from the state. Osteopaths are thus held to be practitioners of medicine, and must stand the examination as required by the laws of the State of Alabama for all persons entering the practice of medicine. This is another victory for the medical laws of Alabama and for the physicians who have labored for a higher medical education and reasonable qualifications to enter on the practice of medicine.

The State of New York could afford to take heed of this wise action of Alabama, which is a model state in its medical organisation and in the administration of its medical laws.

---

THE recent illness of King Edward has been productive of more "newspaper medicine," to which the JOURNAL has so often referred. Some of the Buffalo newspapers were amusingly inaccurate in their descriptions of the disease and operation and the tissues involved. One in particular, after digging deep into the anatomy of the abdomen with unscientific pen, made a really eloquent reference to the "corrective tissue" which was cut through in the course of the operation. For nearly three years the JOURNAL has, in common with medical journals in other cities, advocated the improvement of the daily newspaper by the employment of a member of the staff who shall be competent to deal with scientific matters and prevent such ghastly errors as are constantly creeping into the news and editorial columns. So far as we know there is not a newspaper in this country which has at its service an expert of this character.

---

QUEEN WILHELMINA has decorated the physicians who attended her during her recent grievous illness. Prof. Roosentein, the pathologist of Leyden University, has been promoted to the rank of Commander of the Order of the Lion of the Netherlands, and Drs. Konwer, Roessingh, and Pot have been appointed Knights of the same order.

